# Effects of high‐resistance wheel running on hallmarks of endurance and resistance training adaptations in mice

**DOI:** 10.14814/phy2.15701

**Published:** 2023-06-06

**Authors:** Aurel B. Leuchtmann, Yasmine Afifi, Danilo Ritz, Christoph Handschin

**Affiliations:** ^1^ Biozentrum, University of Basel Basel Switzerland; ^2^ Proteomics Core Facility Biozentrum, University of Basel Basel Switzerland

**Keywords:** endurance exercise, hypertrophy, resistance exercise, skeletal muscle, strength, training adaptation

## Abstract

Exercise effectively promotes and preserves cardiorespiratory, neuromuscular, metabolic, and cognitive functions throughout life. The molecular mechanisms underlying the beneficial adaptations to exercise training are, however, still poorly understood. To improve the mechanistic study of specific exercise training adaptations, standardized, physiological, and well‐characterized training interventions are required. Therefore, we performed a comprehensive interrogation of systemic changes and muscle‐specific cellular and molecular adaptations to voluntary low‐resistance wheel running (Run) and progressive high‐resistance wheel running (RR) in young male mice. Following 10 weeks of training, both groups showed similar improvements in body composition and peak oxygen uptake (*V̇*O_2peak_), as well as elevated mitochondrial proteins and capillarization markers in the *M. plantaris*. Run mice clearly outperformed RR mice in a forced treadmill running capacity test, while RR mice displayed increased grip strength as well as superior mass gains in the *M. soleus*, associated with distinct proteomic changes specifying the two paradigms. Thus, even though both training modalities induce overlapping adaptations, Run interventions preferably improve submaximal running performance, while progressive RR is a valid model to study training‐induced gains in grip strength and plantar flexor hypertrophy.

## Graphical abstract

Young mice were either trained with low‐ or high‐resistance wheel running for 10 weeks or kept sedentary for the same period. Both training groups achieved similar improvements in *V̇*O_2peak_, but low‐resistance running induced a superior adaptation in running capacity. In contrast, grip strength was only elevated following high‐resistance running. The *M. plantaris* and *M. soleus* showed distinct adaptations to both training modalities. The *M. plantaris* displayed a pronounced faster to slower MyHC‐based fiber type shift in both training groups but did not increase in mass. The *M. soleus* hypertrophied in response to both high‐ and low‐resistance wheel running. However, the mass gains were more pronounced with higher resistance, which was also the intervention that substantially shifted fiber type distribution in the *M. soleus*. Tendon‐free muscles were subjected to whole muscle TMT‐LC‐MS/MS, which indicated a similar increase in mitochondrial proteins in the *M. plantaris*, but no difference in the *M. soleus* for these peptides. ECM proteins and structural components of the cytoskeleton and sarcomere appeared to be lowered in the *M. plantaris* by training, while the *M. soleus* seemed to elevate these proteins in a load‐dependent manner.

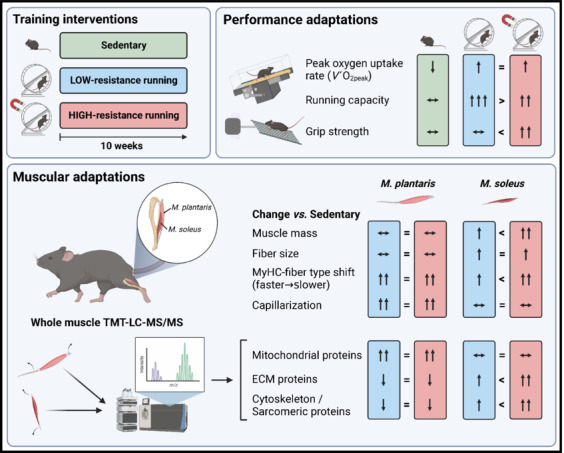

## 1 INTRODUCTION

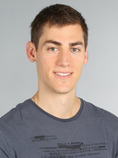

Functional impairments of the musculoskeletal system due to sedentary behavior, aging, or following illness or injury have a major impact on quality of life, morbidity, and mortality. Exercise, in the form of endurance training to promote cardiovascular fitness, and resistance training to promote muscle mass and strength, is effective in preventing a multitude of diseases and disabilities (Egan & Zierath, 2013; Pedersen & Saltin, 2015). However, the mechanisms underlying the multipotent effect of exercise are incompletely understood. To develop exercise‐based and pharmacological therapeutic strategies to improve disability prevention, disuse rehabilitation, and healthy aging, and thereby reduce the socioeconomic burden of associated diseases, an accurate understanding of the molecular underpinnings of exercise‐training adaptation is imperative.

Murine muscle‐specific gain‐ and loss‐of‐function technologies are important tools to provide insights into the factors regulating training adaptations in skeletal muscle in vivo (Verbrugge et al., 2018; Yaghoob Nezhad et al., 2019). However, to identify genes associated with specific phenotypic alterations in response to training, adequate training paradigms for mice are required. Ideally, such interventions recapitulate the desired systemic and skeletal muscle‐specific adaptations while minimizing non‐exercise‐related stress, for example, caused by stimulatory measures in forced settings. Treadmill training, voluntary wheel running and swimming are validated and widely used exercise protocols to study endurance training adaptations in rodents. In contrast, resistance exercise‐based models to investigate muscle growth, increased strength, and other human resistance‐training‐linked muscular remodeling are less well established (Murach et al., 2020). Artificial models include unloading–reloading paradigms or synergist ablation, a surgical model to induce overload and compensatory growth in the spared muscles (Goldberg, 1967; Timson, 1990). However, in recent years, the higher physiological relevance of less invasive and damage‐causing models of skeletal muscle hypertrophy that reflect actual resistance training has been increasingly appreciated (Cui et al., 2020; Murach et al., 2020; Zhu et al., 2021).

Voluntary wheel running represents one of the most stress‐free training interventions for mice (Poole et al., 2020). Activity patterns reflect the physiological rhythms, that is, during the active night phase, and correspond to mice's natural movement pattern of short individual bouts (Manzanares et al., 2018). In the absence of additional load, voluntary wheel running leads to classical endurance training adaptations such as an increase in mitochondrial mass, capillarization, and peak oxygen uptake rate (*V̇*O_2peak_), as well as an oxidative fiber type shift (Poole et al., 2020). Application of resistance to the running wheel, for example by adding a mass or by a magnetic brake, induces muscle hypertrophy and myonuclear accretion in plantar flexor muscles (Dungan et al., 2019; Konhilas et al., 2005; Masschelein et al., 2020). Thus, depending on the load, a shift from endurance to resistance training adaptations can be expected. However, even though loaded wheel running was shown to induce robust cardiac remodeling (Dungan et al., 2019), most of the studies using this paradigm did not assess parameters to quantify training adaptations such as improvements in endurance performance or *V̇*O_2peak_. Moreover, only a few studies performed a direct comparison between low‐load/endurance and high‐load/resistance training (Konhilas et al., 2005; Masschelein et al., 2020). Thus, even though the use of resistance wheel‐running models is increasing, a comprehensive assessment of the continuum between unloaded and resistance wheel‐induced adaptations is still missing. The goal of this study was to provide data on the adaptive range of high‐resistance compared with low‐resistance wheel running (Run) in order to facilitate the selection of an optimal training paradigm for different exercise studies. Therefore, we performed a wide‐ranging interrogation of the systemic, functional as well as muscle‐specific molecular and cellular adaptations to voluntary low‐resistance wheel running and progressive high‐resistance wheel running (RR) in young male mice.

## 2 MATERIALS AND METHODS

### 2.1 Animals

Male C57BL/6J mice were purchased from Janvier Labs (France) and kept in the animal facility of the Biozentrum (University of Basel, Switzerland) at 22°C (range 21–23°C) and under a 12‐h light–dark cycle (6 am to 6 pm) with ad libitum access to regular chow (3432; KLIBA NAFAG) and water. Animals were acclimatized to the facility and individual housing at least 2 weeks before the first measurements took place. The experimental groups were randomized based on the basal assessments, and procedures performed in a blinded manner as far as possible. Training interventions started when mice reached the age of 16 weeks.

### 2.2 Resistance running wheels

During the interventions, mice either had access to a resistance running wheel device (TSE Systems) or were housed in cages equipped with a mock running wheel (created in‐house) to keep the cages of all groups at equal dimensions. The force needed to rotate the wheel was regulated through current‐induced magnetic brakes adjusted in percent (0%–100%). Mice were allowed to familiarize themselves with the wheels (0% resistance) for 5 days before starting the intervention. The running wheel device continuously records wheel movements, which allows the extraction of multiple parameters such as those reported in this study, that is, distance (km), speed (m s^−1^), time spent in the wheel, and number of running bouts. The reported parameters are expressed as average values per daily active phase per week, where one active phase corresponds to the period from 18:00 (lights out) to 09:00 (cessation of running wheel activity). Average work per active phase was calculated as W=τ×θ where θ is the angular displacement in rad (2*π* rad per revolution, that is, covered distance/radius of the wheel [r=5.75cm]) and τ the torque necessary to maintain wheel speed at a given resistance. By applying increasing levels of current to the electromagnet of a resistance running wheel (*n* = 1), TSE Systems provided a torque‐current‐curve to determine the torque at a given percent of resistance. The highest electromagnetic resistance (100%) was achieved with a current of 223 mA.

### 2.3 Training interventions

In the weeks prior to starting the experiment, baseline values for body mass, endurance parameters (*V̇*O_2peak_ and treadmill running capacity), grip strength, and body composition (lean and fat mass) were measured and used for balanced group selection. All parameters were retested at the end of the intervention period. For the training intervention, 24 mice were equally divided into a sedentary control group with a mock running wheel (Sed; *n* = 8), a running wheel group with access to a wheel with low (50%) resistance (Run; *n* = 8) and a high‐resistance running group with access to a wheel with progressively increased resistance (RR; *n* = 8). For all mice in RR, resistance was 50% in the first, 60% in the second, 65% in the third, and 68% in the fourth week. During Weeks 5–10, resistance was adjusted for each mouse individually. Specifically, resistance was progressively increased in steps of 2% every day until a mouse reached the minimum threshold of 2 km per daily active phase. In case a mouse fell below this threshold for two consecutive active phases, resistance was reduced by 2% for two active phases and then raised again by 2% once the mouse reached 2 km.

### 2.4 Body composition analysis

Body composition (including lean and fat mass) was assessed with an EchoMRI‐100 analyzer (EchoMRI Medical Systems) in minimally restrained, conscious mice. Measurements were taken before (baseline) and at the end of the intervention.

### 2.5 Grip strength in vivo

Muscle force was estimated in vivo by measuring the peak force of the whole limb grip (i.e., fore and hind limbs together) using a Grip Strength Meter (Chatillon; Columbus Instruments). Before the first test, mice were familiarized by performing four rounds of three consecutive pulls with at least a 15‐min break between rounds. For a pull, the animal was held by the base of its tail and placed with all four limbs on the angled mesh pull bar assembly connected to a force transducer. Once the mouse gripped firmly with all four limbs, it was pulled at a consistent speed horizontally away from the sensor until the grip was released. On testing days, three consecutive pulls were performed with the best value noted (in kilogram‐force; 1 kgf = 9.806650 N). This procedure was performed a total of four times with at least a 10‐min break between rounds. The mean of all four rounds was normalized to body mass.

### 2.6 Evaluation of running capacity (treadmill exhaustion test)

Mice were subjected to a running capacity test before (baseline) and at the end of the intervention using a six‐lane open treadmill system (Columbus Instruments). Before the baseline test, mice were familiarized with the treadmill for two consecutive days. On Day 1, mice were placed on the static treadmill band for 5 min followed by running at 5, 8, and 10 m min^−1^ for 5 min each at 5° inclination. On Day 2, mice were running at 5, 8, 10, and 12 m min^−1^ for 5 min each at 5° inclination. On the testing day (2 days after familiarization), mice were weighed and basal values for blood lactate (Lactate Plus meter, Nova Biomedical) were measured. Maximum running capacity was then evaluated by letting the mice run to exhaustion on an open treadmill with 5° inclination using an incremental step protocol: 5 min at 5 m min^−1^, 3 min at 8 m min^−1^ followed by a 2 m min^−1^ increase every 3 min until exhaustion. Exhaustion was determined by the animal failing to remain on the treadmill belt despite a mild electric stimulus (0.5 mA, 200 ms pulse, 1 Hz) and prodding. At this point, the animal was taken off the treadmill to determine blood lactate levels immediately. For the retest, the same protocol was used but from 38 m min^−1^ (the maximum speed reached by mice in the baseline test) speed was increased only every 10 min by 2 m min^−1^. This was done to better assess potential performance differences between the two running groups. All tests were performed in the morning (lights on) after the food had been removed for at least 2 h and mice were randomly assigned to groups of four.

### 2.7 Ramp‐sprint test to determine *V̇*O_2peak_

*V̇*O_2peak_ was measured using a closed Metabolic Modular Treadmill system (Columbus Instruments). Mice were subjected to a short, high‐intensity ramp‐sprint protocol optimized to determine *V̇*O_2peak_ as previously described (Leuchtmann et al., 2022) with minor modifications (Figure 2a). In brief, after an 8‐min resting measurement followed by a 3‐min warm‐up at 8 m min^−1^, speed was continuously increased by 0.04 m min^−1^ s^−1^ (i.e., slowly ramped up). Slope was set to 15°. Stainless steel grids at the end of each lane provided a mild electrical stimulus (0.5 mA, 200 ms pulse, 1 Hz) to keep the mice running. Mice ran until *V̇*O_2_ values plateaued and/or if the animal remained on the electrical grid without any attempt to go back on the treadmill belt. Before being placed on the treadmill, mice were weighed, and lactate values (Lactate Plus meter, Nova Biomedical) were determined via tail blood. Lactate values were measured again immediately after mice met the above‐described abortion criteria. For the tests, mice were randomly assigned to groups of two, and food was removed at least 1 h before the test. A specific gas blend (Primary Standard Grade; 20.5% O_2_ and 0.5% CO_2_ in N_2_) was used to calibrate the span or gain of both the O_2_ and CO_2_ sensors of the Oxymax system before each test session. The flow was set to 0.6 L min^−1^. *V̇*O_2_ and *V̇*CO_2_ were measured every 5 s.

### 2.8 Sample collection

Running wheels were blocked 24 h before tissue collection, which was performed between 8 and 12 am on six mice on four consecutive days. To minimize variation in food intake, food was removed after the light has turned on (6 am) for all mice that had to undergo dissection on a given dissection day. Mice were sacrificed by gradual CO_2_ asphyxiation and left and right *M. quadriceps femoris* (QUAD), *M. tibialis anterior* (TA), *M. gastrocnemius* (GAS), *M. soleus* (SOL), *M. plantaris* (PLA), *M. triceps brachii* (TRI), and *M. biceps brachii* (BIC) and *M. brachioradialis* (BR) were harvested and weighed. Muscles of the right hind and fore limb were frozen in optimal cutting temperature medium (Tissue‐Tek O.C.T™) at resting length and snap‐frozen in liquid nitrogen‐cooled isopentane (around −145°C) before transfer to liquid nitrogen and storage at −80°C for histochemical analyses. Muscles of the left hind and fore limb were snap‐frozen in liquid nitrogen and stored at −80°C for protein extraction.

### 2.9 Immunostaining of muscle cross sections

Serial muscle sections (10 μm) were cut from the mid‐belly at −22°C on a cryostat (Leica, CM1950), collected on Fisherbrand Superfrost/Plus adhesion slides (epredia, Gerhard Menzel GmbH), and kept at −80°C until further use. *Fiber typing*: Sections were rehydrated and blocked in Dulbecco's phosphate‐buffered saline (PBS, Sigma) containing 10% goat serum (Sigma) and 0.4% Triton‐X 100 (Sigma) for 30 min followed by 1‐h incubation at RT with a primary antibody cocktail of mouse IgG2b monoclonal anti‐MyHC‐I (1:50; BA‐D5), mouse IgG1 monoclonal anti‐MyHC‐IIA (1:200; SC‐71), mouse IgM monoclonal anti‐MyHC‐IIB (1:100; BF‐F3) from the Developmental Studies Hybridoma Bank (DHSB), and a rabbit polyclonal antibody to laminin (1:154; Abcam # ab11575) and 10% goat serum. Sections were washed 3 × 5 min in PBS followed by 1‐h incubation (at RT and in the dark) with the following secondary antibody cocktail: DyLight 405 Goat anti‐Mouse (GαM) IgG (1:400; 115‐475‐207, Jackson ImmunoResearch), AF568 IgG1 GαM IgG1 (1:1000; A‐21124, Life Technologies), AF488 IgM GαM IgM (1:1000; A‐21042, Life Technologies), and AF647 Donkey anti‐Rabbit IgG (1:2000; 711‐605‐152, Jackson ImmunoResearch). Sections were washed 3 × 5 min with PBS and dehydrated in EtOH baths of increasing concentration (2 × 2 min 70% EtOH and 2 × 2 min 100% EtOH). Sections were air‐dried, coverslipped using ProLong Gold Antifade Reagent (Invitrogen, P36930) as mounting medium, and stored at 4°C for 12 h before imaging. *NADH staining*: Sections were allowed to reach 37°C before exposing them for exactly 20 min at 37°C to the staining solution (1 Nitro Blue Tetrazolium tablet (Sigma N5514) dissolved in 10 mL PBS + 10.6 mg β‐nicotinamide adenine dinucleotide [Sigma N8129]). Sections were washed 3 × 3 min in deionized water (at RT) and dehydrated in EtOH baths of increasing concentration and a final xylene bath (2 min 70% EtOH, 2 min 95% EtOH, 2 min 100% EtOH, and 2 min 100% Xylene). Sections were air‐dried, coverslipped using Histomount Mounting Solution (Invitrogen 008030) as mounting medium, and stored at 4°C before imaging.

### 2.10 Slide imaging and image analyses

Muscle sections were imaged at the Biozentrum Imaging Core Facility with an Axio Scan.Z1 Slide Scanner (Zeiss) equipped with appropriate band‐pass filters. Analysis of fiber type was performed on one randomly chosen muscle section per mouse (folded or torn sections were excluded). The ImageJ software (NIH) and in‐house developed Fijii macros allowed an automated analysis of muscle fiber types (based on intensity thresholds) and fiber size (i.e., cross‐sectional area [CSA]; based on cell segmentation by laminin) (Delezie et al., 2019). This resulted in the analysis of 995 ± 175 (Sed), 934 ± 133 (Run), and 942 ± 125 (RR) fibers for the *M. plantaris* and 957 ± 159 (Sed), 858 ± 173 (Run), and 985 ± 292 (RR) fibers for the *M. soleus*.

The NADH staining resulted in muscle fibers rich in NADH‐linked flavoprotein activity staining dark blue and densely granulated, and muscle fibers with low levels of NADH staining light blue and sparsely granulated. Quantification of slides was performed as previously described (Leuchtmann et al., 2020). In brief, on stained sections, three ranges of fiber staining intensity can be identified by eye. Fibers of a given section were thus classified by eye according to their relative staining intensity as fibers showing high (strong, i.e., darkest straining), intermediate (intermediate staining), or no (weakest or no staining) NADH levels.

### 2.11 Protein extraction

Frozen, powdered muscle tissue was transferred to 2 mL tubes containing a cold metal bead (Qiagen, stainless steel 5 mm [200 pc]). 400 and 200 μL ice cold radioimmunoprecipitation assay (RIPA) buffer (150 mM NaCl, 1% v/v Nonidet‐P40 substitute, 0.2% v/v Na‐deoxycholate, 0.1% v/v SDS, 50 mM TRIS–HCl [pH 7.5], 1 mM EDTA, 1 mM DTT, 10 mM nicotinamide) and freshly added protease inhibitors (cOmplete mini EDTA free, Roche) was added to total left *M. plantaris* and *M. soleus*, respectively, and homogenized for 10 s at 5 m s^−1^ using an MP FastPrep bead homogenizer. Subsequently, tubes were centrifuged at 2000 ***g*** for 1 min to remove bubbles and then incubated for 30 min at 4°C with rotation. Lysates were then transferred to 1.5 mL tubes and sonicated for 10 min (10 cycles of 30 s/30 s ON/OFF) using a Bioruptor® Pico followed by centrifugation at 16,000 ***g*** for 10 min at 4°C. The resulting supernatant was transferred to a fresh tube and protein concentration was measured by the bicinchoninic acid protein assay.

### 2.12 Protein digestion for mass spectrometry analysis

One hundred micrograms of protein per sample was diluted in RIPA buffer and SDS to reach a final concentration of 5% SDS and a volume of 25 μL. Proteins were alkylated in 20 mM Iodoacetamide for 30 min and were digested using S‐Trap™ micro spin columns (Protifi) according to the manufacturer's instructions. Shortly, 12% phosphoric acid was added to each sample (final concentration of phosphoric acid 1.2%) followed by the addition of S‐trap buffer (90% methanol, 100 mM TEAB [pH 7.1]) at a ratio of 6:1. Samples were mixed by vortexing and loaded onto S‐trap columns by centrifugation at 4000 ***g*** for 1 min followed by three washes with S‐trap buffer. Digestion buffer (50 mM TEAB [pH 8.0]) containing sequencing‐grade modified trypsin (1/25, w/w; Promega) was added to the S‐Trap column and incubated for 1 h at 47°C. Peptides were eluted by the consecutive addition and collection by centrifugation at 4000 ***g*** for 1 min of 40 μL digestion buffer, 40 μL of 0.2% formic acid, and finally 35 μL 50% acetonitrile containing 0.2% formic acid. Samples were dried under vacuum and stored at −20°C until further use.

### 2.13 TMT labeling and LC‐MS/MS analysis of whole *M. plantaris* and *M. soleus*

Sample aliquots comprising 12.5 μg of peptides were labeled with isobaric tandem mass tags (TMTpro 16‐plex, Thermo Fisher Scientific). Peptides were resuspended in 10 μL labeling buffer (2 M urea, 0.2 M HEPES, pH 8.3) by sonication, and 2.5 μL of each TMT reagent were added to the individual peptide samples followed by a 1‐h incubation at 25°C shaking at 500 rpm. To quench the labelling reaction, 0.75 μL aqueous 1.5 M hydroxylamine solution was added and samples were incubated for 5 min at 25°C shaking at 500 rpm followed by pooling of all samples. The pH of the sample pool was increased to 11.9 by adding 1 M phosphate buffer (pH 12) and incubated for 20 min at 25°C and 500 rpm shaking to remove TMT labels linked to peptide hydroxyl groups. Subsequently, the reaction was stopped by adding 2 M hydrochloric acid until a pH <2 was reached. Finally, peptide samples were further acidified using 5% TFA, desalted using BioPureSPN MACRO™ SPE cartridges (Nest group) according to the manufacturer's instructions, and dried under vacuum.

TMT‐labeled peptides were fractionated by high‐pH reversed‐phase separation using an XBridge Peptide BEH C18 column (3.5 μm, 130 Å, 1 mm × 150 mm, Waters) on an Ultimate 3000 system (Thermo Scientific). Peptides were loaded on a column in buffer A (20 mM ammonium formate in water, pH 10) and eluted using a two‐step linear gradient from 2% to 10% in 5 min and then to 50% buffer B (20 mM ammonium formate in 90% acetonitrile, pH 10) over 55 min at a flow rate of 42 μL min^−1^. Elution of peptides was monitored with a UV detector (215 nm, 254 nm), and a total of 36 fractions was collected, pooled into 12 fractions using a postconcatenation strategy as previously described (Wang et al., 2011), and dried under vacuum.

Dried peptides were resuspended in 0.1% aqueous formic acid and subjected to LC‐MS/MS analysis using an Orbitrap Eclipse Tribrid Mass Spectrometer fitted with Ultimate 3000 nanosystem and a FAIMS Pro interface (all Thermo Fisher Scientific) and a custom‐made column heater set to 60°C. Peptides were resolved using an RP‐HPLC column (75 μm × 30 cm) packed in‐house with C18 resin (ReproSil‐Pur C18–AQ, 1.9 μm resin; Dr. Maisch GmbH) at a flow rate of 0.3 μL min^−1^. The following gradient was used for peptide separation: from 2% B to 12% B over 5 min to 30% B over 70 min to 50% B over 15 min to 95% B over 2 min followed by 18 min at 95% B then back to 2% B over 2 min followed by 8 min at 2% B. Buffer A was 0.1% formic acid in water and buffer B was 80% acetonitrile, 0.1% formic acid in water.

The mass spectrometer was operated in DDA mode with a cycle time of 3 s between master scans. Throughout each acquisition, the FAIMS Pro interface switched between CVs of −40 and −70 V with cycle times of 1.5 and 1.5 s, respectively. MS1 spectra were acquired in the Orbitrap at a resolution of 120,000 and a scan range of 400–1600 *m*/*z*, AGC target set to “Standard,” and maximum injection time set to “Auto.” Precursors were filtered with precursor selection range set to 400–1600 *m*/*z*, monoisotopic peak determination set to “Peptide,” charge state set to 2–6, a dynamic exclusion of 45 s, a precursor fit of 50% in a window of 0.7 *m*/*z*, and an intensity threshold of 5e3.

Precursors selected for MS2 analysis were isolated in the quadrupole with a 0.7 *m*/*z* window and collected for a maximum injection time of 35 ms with the AGC target set to “Standard.” Fragmentation was performed with a CID collision energy of 30% and MS2 spectra were acquired in the IT at the scan rate “Turbo.” MS2 spectra were subjected to RTS using a mouse database containing 17,093 entries downloaded from Uniprot on 20,220,222 using the following settings: enzyme was set to “Trypsin”, TMTpro16plex (K and N‐term), and Carbamidomethyl (C) was set as fixed modification, Oxidation (M) was set as variable modifications, maximum missed cleavages were set to 1 and maximum variable modifications to 2. Maximum search time was set to 100 ms, the scoring threshold was set to 1.4 XCorr, 0.1 dCn, 10 ppm precursor tolerance, charge state 2, and “TMT SPS MS3 Mode” was enabled. Subsequently, spectra were filtered with a precursor selection range filter of 400–1600 *m*/*z*, precursor ion exclusion set to 25 ppm low and 25 ppm high, and isobaric tag loss exclusion set to “TMTpro.” MS/MS product ions of precursors identified via RTS were isolated for an MS3 scan using the quadrupole with a 2 *m*/*z* window and ions were collected for a maximum injection time of 200 ms with a normalized AGC target set to 200%. SPS was activated and the number of SPS precursors was set to 10. Isolated fragments were fragmented with normalized HCD collision energy set to 55% and MS3 spectra were acquired in the orbitrap with a resolution of 50,000 and a scan range of 100–500 *m*/*z*.

The acquired raw files were analyzed using the SpectroMine software (Biognosis AG). Spectra were searched against a murine database consisting of 17,093 protein sequences (downloaded from Uniprot on February 22, 2022). Standard Pulsar search settings for TMT 16 pro (“TMTpro_Quantification”) were used and resulting identifications and corresponding quantitative values were exported on the PSM level using the “Export Report” function. Acquired reporter ion intensities were employed for automated quantification and statistical analysis using the in‐house developed SafeQuant R script (v2.3) (Ahrné et al., 2016). This analysis included adjustment of reporter ion intensities, global data normalization by equalizing the total reporter ion intensity across all channels, data imputation using the knn algorithm, summation of reporter ion intensities per protein and channel, and calculation of protein abundance ratios. To meet additional assumptions (normality and homoscedasticity) underlying the use of linear regression models and *t*‐tests, MS‐intensity signals were transformed from the linear to the log scale. The summarized protein expression values were used for statistical testing of between condition differentially abundant proteins. Here, empirical Bayes‐moderated *t*‐tests were applied, as implemented in the R/Bioconductor limma package (http://bioconductor.org/packages/release/bioc/html/limma.html). Significance was defined as *p* < 0.01 for *M. plantaris* and *p* < 0.05 for *M. soleus*.

Overlap between different datasets was determined with Venny (version 2.1, https://bioinfogp.cnb.csic.es/tools/venny/), and Venn's diagrams for visualization were drawn with the R Shiny app VennDiagrams (run on venddigrams.net until May 2022). Protein enrichment analyses were performed with the Metascape gene annotation and analysis resource (Zhou et al., 2019) including heatmaps for the visualization of enriched pathways, Circos plots to visualize protein‐ and/or ontology‐based overlaps between protein lists and transcriptional regulatory relationships unraveled by sentence‐based text‐mining (TRRUST) enrichment analyses. Protein–protein interaction networks generated by Metascape were optimized for visualization using Cytoscape (version 3.9.1). Heatmaps to visualize the enrichment of selected proteins were created using Morpheus (https://clue.io/morpheus).

### 2.14 Immunoblotting

The same protein extracts as used for mass spectrometry analysis were diluted in RIPA buffer and 4X Laemmli Sample Buffer (Bio‐Rad) with 2‐mercaptoethanol (Sigma) and boiled for 5 min at 50°C as recommended for OXPHOS blots. Equal amounts of protein (20 μg) were separated on 4%–20% Mini‐Protean TGX Precast Protein Gels (Bio‐Rad) and transferred on nitrocellulose membranes (Amersham Protran 0.45 NC, 10600007, 0.45 μm). Membranes were blocked for 1 h in 5% milk in Tris‐buffered saline + 0.1% Tween 20 (TBS‐T) before overnight incubation at 4°C and shaking with the following antibodies: Total OXPHOS Rodent WB Antibody Cocktail (1:1000 in 1% milk, PBS, ab110413, Abcam) and CD31 (1:1000 in 5% BSA, TBS‐T, ab28364, Abcam). After washing, membranes were incubated with peroxidase‐conjugated secondary antibodies (1:10,000 in TBS‐T, Dako) for 1 h at RT, followed by antibody binding detection using chemiluminescence horseradish peroxidase substrate detection kits (ECL, Supersignal West Dura or Femto; Pierce) and a Fusion FX imager (Viliber). Quantification was done with the Fusion FX software. Relative protein levels were determined by normalizing the band intensity of the target to the loading control (Ponceau S stain) and the average of the control group (Sed) within one gel.

### 2.15 Statistical analyses

Data are presented as mean ± SD including individual values where indicated or as box and whisker plots. In the latter, boxes depict the 25th and 75th percentiles (upper and lower perimeters), the median (midline), and the mean (cross). Whiskers are plotted based on the interquartile range (IQR), that is, the difference between the 25th and 75th percentile. The upper whisker is drawn to the largest value in the dataset that is smaller than (or equal to) the 75th percentile plus 1.5 times IQR (upper fence) and the lower whisker is drawn to the smallest value in the dataset that is greater than (or equal to) the 25th percentile minus 1.5 times IQR (lower fence). Any values greater than the upper or smaller than the lower fence are plotted as individual points. Data were represented and analyzed using the GraphPad Prism 8.0 software (GraphPad Software). The *n* number used for each experiment is indicated in the figure legends. Dropout curves of the distance ran (m) during the running capacity test were plotted as Kaplan–Meier plots and were compared using a Mantel–Cox log‐rank test. Statistical significance was tested by Student's *t*‐tests for pairwise comparisons, while one‐way ANOVAs with Fisher's LSD post hoc tests were used to compare between three groups, so long as the ANOVA reached statistical significance. Two‐way ANOVAs or two‐way repeated measures ANOVAs for multiple recordings over time, with Sidak's or Tukey's post hoc tests to compare between groups with two independent variables. The level of significance was set at *p* < 0.05 and significant differences and trends (0.05 ≤ *p* ≤ 0.08) are reported where appropriate.

## 3 RESULTS

### 3.1 Characteristics of the training stimulus provided by Run and RR and their effect on body composition

**FIGURE 1:**
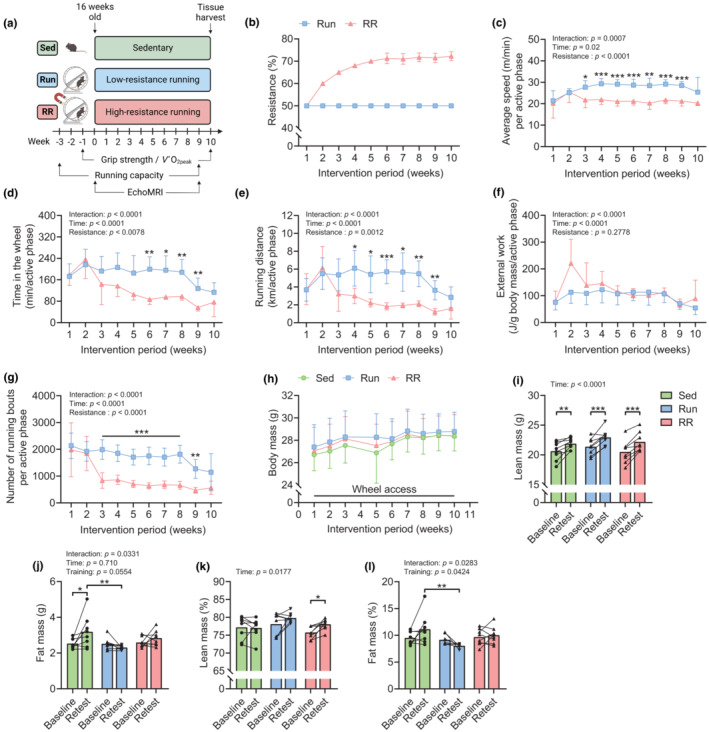
Characterization of the training stimulus provided by Run and RR and their effect on body composition. (a) Graphical illustration of the experimental approach of wheel running interventions and timing of baseline measurements and retests. (b) Average resistance, (c) running speed, (d) time spent in the wheel, (e) running distance, (f) external work, and (g) number of running bouts per active phase throughout the study. (h) Body mass trajectory for all groups from start (Week 1) to the end (Week 10) of the intervention period. (i) Body lean mass, (j) fat mass, (k) body lean mass percentage, and (l) body fat mass percentage at baseline and after 9 weeks. Data are presented as mean ± SD (b–h), mean and individual paired values connected with a black line (i–l), *n* = 8 for all datasets. Two‐way repeated‐measure ANOVAs followed by Sidak's or Tukey's post hoc tests (c–l). *p < 0.05, **p < 0.01, ***p < 0.001. Scheme in (a) was created with BioRender.com.

To compare the adaptations to low‐load wheel running and progressive resistance wheel running, mice received access to running wheels set to 50% (low) resistance (Run) or a progressive increase in resistance (RR) for 10 weeks (Figure 1a). For all mice in RR, resistance was 50% in the first, 60% in the second, 65% in the third, and 68% in the fourth week, after which resistance was adjusted for each mouse individually (Figure 1b). With the set minimum threshold (2 km per daily active phase), mice maintained resistances between 68% and 74% during Weeks 6–10. The progressive increase in load in RR reduced running speed, time in the wheel, running distance, and a number of running bouts from week three onwards (Figure 1b–g). The resulting external work adjusted for body mass was equal in both running groups throughout the study (Figure 1f). All groups showed similar body mass trajectories, characterized by a continuous rise (Figure 1h). While this was mainly driven by a gain in lean mass in trained mice, sedentary mice also significantly accumulated fat mass (*p* = 0.0173) (Figure 1i,j). Overall, this tissue remodeling increased body lean mass percentage from baseline in RR (*p* = 0.0434) (Figure 1k) and a higher body fat percentage in sedentary control animals (Sed) compared to Run mice in the retest (*p* = 0.0012) (Figure 1l).

### 3.2 Functional adaptations to 10 weeks of Run and RR

**FIGURE 2:**
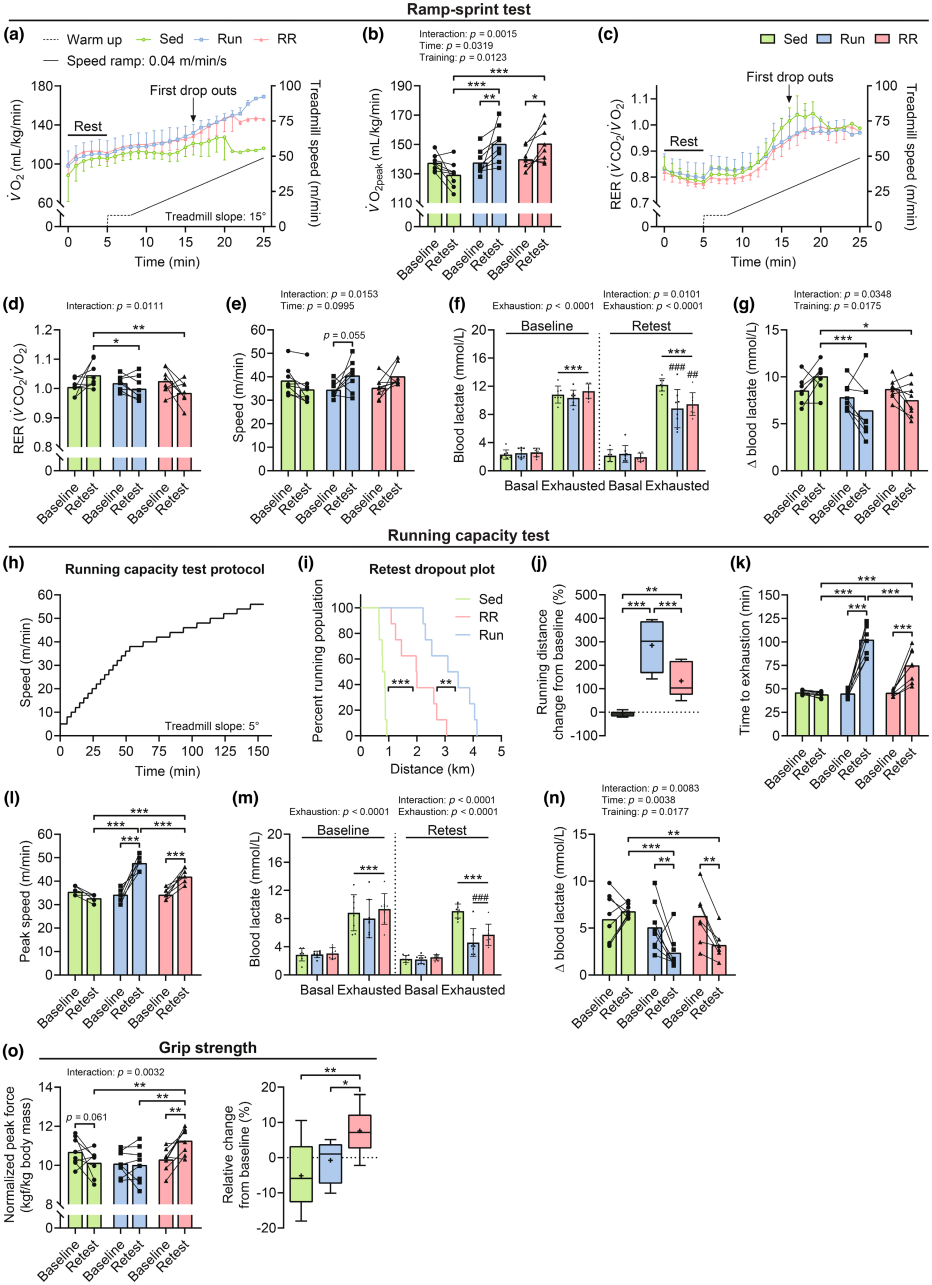
Training‐induced adaptations in oxygen consumption rates, running performance, and grip strength. Indirect calorimetry measurements at rest and during a ramp‐sprint protocol on a closed treadmill system with a 15° slope: (a) O_2_ consumption rates and (c) RER (*V̇*CO_2_/*V̇*O_2_) as a function of speed. (b) Peak oxygen uptake (*V̇*O_2peak_), (d) RER, and (e) speed values at *V̇*O_2peak_ at baseline and after 10 weeks (Retest) of the intervention. (f) Blood lactate levels before (Basal) and directly after (Exhausted) the ramp‐sprint test and (g) expressed as the difference exhausted‐basal (Δ) at baseline and after 10 weeks (Retest). (h) Incremental step protocol used to asses running performance: (i) Kaplan–Meier plot of distance covered in the retest, (j) change from baseline in the running distance in percent. (k) Time to exhaustion (min) and (l) peak speed (m min^−1^) at baseline and after 9 weeks (Retest). (m) Blood lactate levels before (Basal) and after (Exhausted) the running capacity test and (n) expressed as the difference exhausted‐basal (Δ) at baseline and after 9 weeks (Retest). (o) Normalized in vivo muscle force estimated by measuring the peak force (kgf, kilogram‐force) of the whole limb grip (left panel) and relative change from baseline (right panel). Data are presented as mean ± SD (a, c, f, m), mean and individual paired values connected with a black line (b, d, e, g, k, l, o), or as Tukey's box and whisker plots (j, o). For all datasets, *n* = 8 except for Sed in (f, g) where one data point was removed at “baseline‐exhausted” due to a technical error. Two‐way repeated‐measure ANOVAs followed by Sidak's or Tukey's post hoc tests (b, d–g, k–o), Mantel‐Cox log‐rank (i), and one‐way ANOVA with Fisher's LSD (j and right panel in n). *p < 0.05, **p < 0.01, ***p < 0.001, ^##^ *p* < 0.01, and ^###^ *p* < 0.01 Sed vs. Run or RR of the same time point.

To assess the slope of oxygen uptake rate (*V̇*O_2_) during exercise and to determine changes in peak oxygen uptake (*V̇*O_2peak_) in response to training, mice were subjected to a short, intense ramp‐sprint protocol on a closed treadmill system to acquire real‐time respiratory measurements using indirect calorimetry before and after 10 weeks of training. Figure 2a,c shows the retest curves of *V̇*O_2_ and RER (*V̇*CO_2_/*V̇*O_2_), respectively, during rest and the speed ramp until exhaustion. In trained mice, *V̇*O_2_ values increased linearly as speed accelerated until the point of failure, whereas in the sedentary group, *V̇*O_2_ was only elevated close to exhaustion (Figure 2a). *V̇*O_2peak_ was augmented by training in both running groups (Run, *p* = 0.0095; RR, *p* = 0.0337) and remained constant in sedentary mice during the same period (Figure 2b). As a result, retest *V̇*O_2peak_ values of both training groups were significantly higher compared to sedentary mice (Sed vs. Run and Sed vs. RR, *p* = 0.0002) (Figure 2b). The RER depicting substrate preference during exercise showed similar trajectories for all groups at rest and at lower speeds following the training intervention (Figure 2c). In contrast, close to exhaustion, RER values rose to a larger extent in sedentary mice (Figure 2c) and peaked significantly higher in the sedentary group compared to the two trained groups at *V̇*O_2peak_ (Sed vs. Run, *p* = 0.0458; Sed vs. RR, *p* = 0.0059) (Figure 2d). Overall, both running groups showed similar training‐induced improvements in *V̇*O_2peak_, RER, and peak speed (Figure 2b,d,e) and exhibited lower blood lactate concentrations (Sed vs. Run, *p* = 0.0001; Sed vs. RR, *p* = 0.0019) (Figure 2f) and delta lactate (difference exhausted‐basal) values (Sed vs. Run, *p* = 0.0008; Sed vs. RR, *p* = 0.0226) (Figure 2g) in the retest.

**FIGURE 3:**
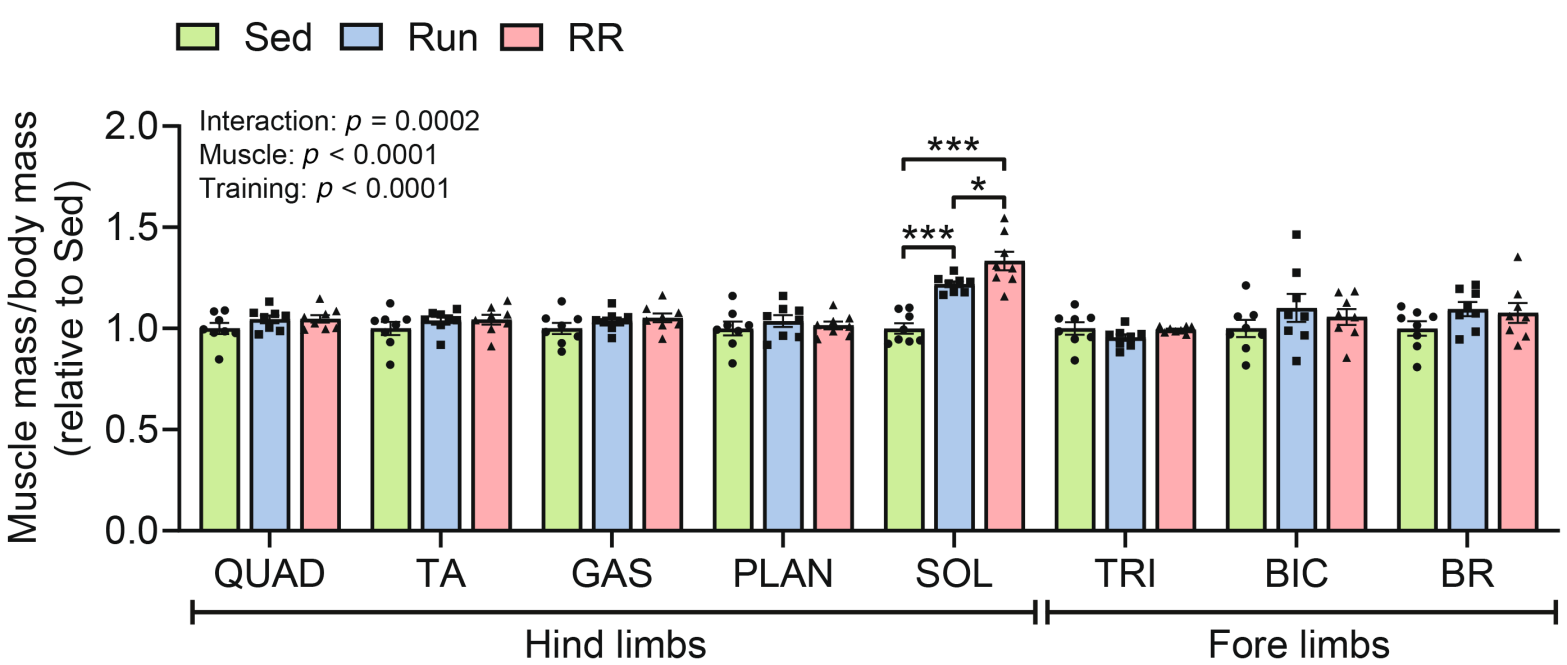
Changes in individual muscle masses. Masses of various fore and hind limb muscles expressed as fold change relative to Sed after normalization to body mass (*n* = 8). QUAD, *M. quadriceps femoris*; TA, *M. tibialis anterior*; SOL, *M. soleus*; GAS, *M. gastrocnemius*; PLAN, *M. plantaris*; TRI, *M. triceps brachii*; BIC, *M. biceps brachii* and BR, *M. brachio radialis*. Data are presented as mean ± SD including individual values. Two‐way ANOVA followed by Tukey's post hoc test. *p < 0.05, ***p < 0.001.

To estimate whether there are differences in fractional utilization, that is, the proportion of *V̇*O_2peak_ that can be sustained at lower intensities and during prolonged exercise, we subjected the same mice to an extended endurance test protocol (Figure 2h). Strikingly, despite the similar *V̇*O_2peak_ values (Figure 2b), Run mice significantly outperformed RR mice in the retest (*p* = 0.0075). (Figure 2i) as they improved by nearly 300% in distance compared to the 120% achieved by the RR group (Run vs. RR, *p* = 0.0007) (Figure 2j). Similarly, time to exhaustion (*p* < 0.0001) (Figure 2k) and peak speed (*p* < 0.0001) (Figure 2l) differed significantly between the two running groups. The sedentary group maintained the level of performance in this test, thus covering the same distance and reaching similar speeds before and after the observation period (Figure 2j–l). Collectively, this suggests that the changes in *V̇*O_2peak_ observed in this cohort are not strongly determining submaximal performance levels. Consistent with the results from the ramp‐sprint test, trained mice accumulated significantly less lactate during the retest compared to baseline and to sedentary mice (Figure 2m,n). This indicates that both training modalities led to metabolic remodeling with reduced lactate production and/or higher clearance. A hallmark of functional adaptation to resistance training is a gain in muscle strength. Grip strength measurements in vivo revealed that only resistance running induced an increase in grip strength in response to training (*p* = 0.0074), leading to greater retest values compared to those of sedentary and Run mice (Sed vs. RR, *p* = 0.0079; Run vs. RR, *p* = 0.0034) (Figure 2o). Finally, among several hind and fore limb skeletal muscles, the *M. soleus* was the only one that showed an increase in mass in response to training (Sed vs. Run and Sed vs. RR, *p* < 0.0001) and this in a load‐dependent manner (Run vs. RR, *p* = 0.0307) (Figure 3).

### 3.3 Adaptations of the *M. plantaris* in mice subjected to 10 weeks of Run or RR

**FIGURE 4:**
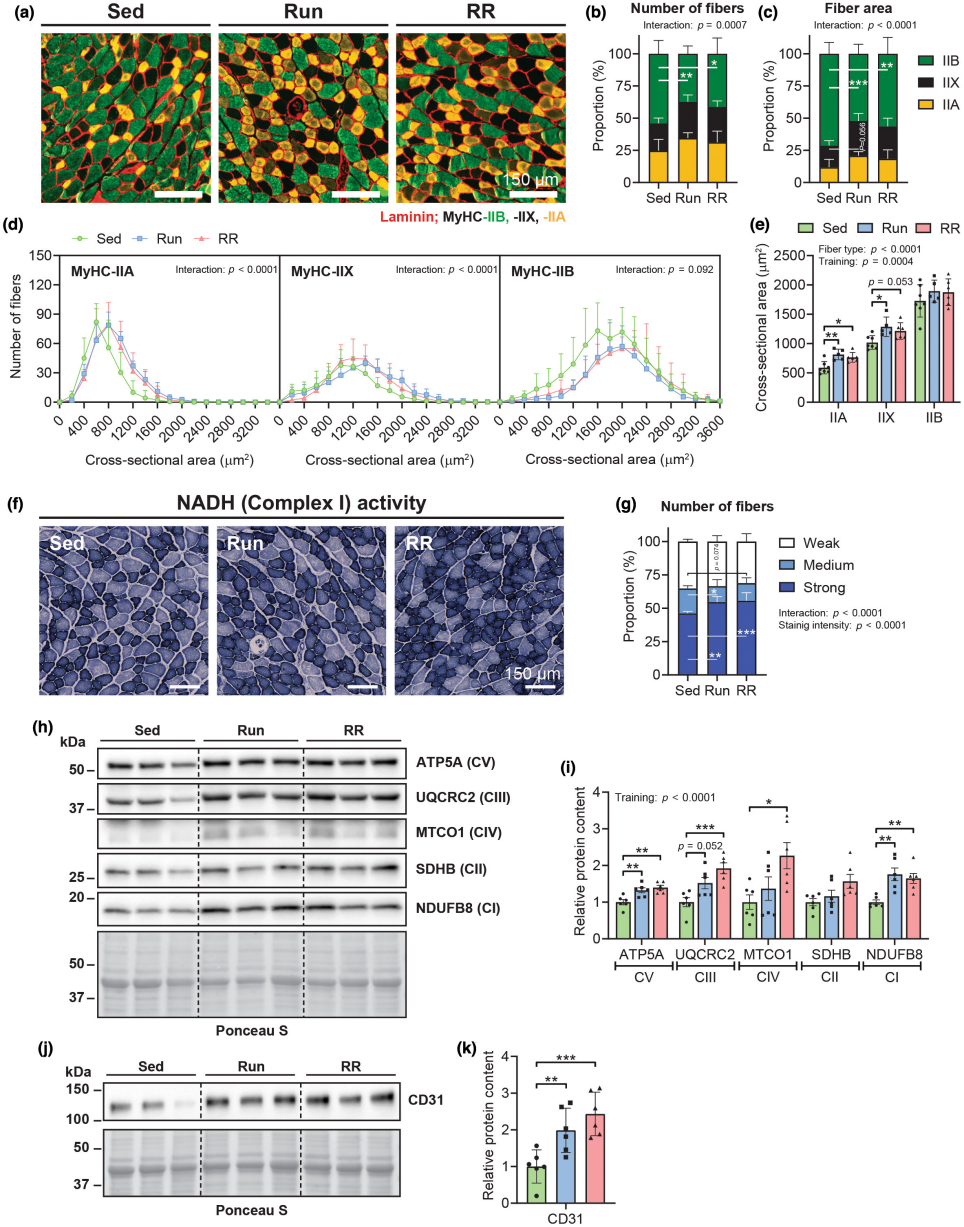
Cellular and molecular adaptations of the *M. plantaris* following Run and RR. (a) Representative magnifications of whole‐muscle cross‐sectional images from the *M. plantaris* stained with antibodies against MyHC‐IIA (yellow), ‐IIB (green) as well as laminin (red), while fibers without staining were classified as MyHC‐IIX. Quantification of the MyHC‐isoform‐based fiber‐type distribution as a proportion of (b) the total number of fibers and (c) the total cross‐sectional area (CSA) occupied by fibers. (d) Frequency histograms for the individual fiber types display the number of fibers that had the indicated CSA (μm^2^). (e) Mean CSA of MyHC‐IIA, ‐IIX, and ‐IIB fibers. (b–e) Sed, *n* = 7; Run, *n* = 5; RR, *n* = 6. (f) Representative images of NADH enzyme activity staining and (g) resulting proportion of fibers quantified as fibers showing weak, medium, or strong NADH activity (Sed, *n* = 6; Run, *n* = 5; RR, *n* = 6). Western blots of mitochondrial respiratory chain complexes (c) I–V (h) and endothelial cell marker CD31 (j) along with the band quantifications of two blots per protein target (i and k) (*n* = 6). Data are presented as mean ± SD including individual values where indicated. Two‐way ANOVAs with Tukey's post hoc test (b–e, g and i) and one‐way ANOVA with Fisher's LSD in (k). *p < 0.05, **p < 0.01, ***p < 0.001.

Based on the diverging performance in the running capacity test despite similar *V̇*O_2peak_ values, we checked whether the two training protocols had differential effects on fiber‐type composition in the *M. plantaris*. Both running groups exhibited a myosin heavy chain (MyHC)‐based slow‐twitch fiber‐type shift characterized by a reduction in MyHC‐IIB positive fibers (Sed vs. Run, *p* = 0.0019, Sed vs. RR, *p* = 0.0138) (Figure 4a,b). As a result, MyHC‐IIB fibers occupied a much larger proportion of the total fiber CSA in Sed mice compared to both exercise groups (Sed vs. Run, *p* = 0.0002, Sed vs. RR, *p* = 0.0018) (Figure 4c). The shift in MyHC‐isoforms led to a shift in fiber size distribution, where MyHC‐IIA and ‐IIX fibers had larger CSAs in both training groups (Figure 4d,e) but without affecting overall muscle mass (Figure 3). Activity stainings of the mitochondrial complex I electron donor NADH revealed an increase in the proportion of oxidative fibers with strong activity at the cost of more glycolytic fibers with intermediate activity in both training groups (Figure 4f,g), thereby mirroring the metabolic and the contractile fiber‐type shifts. The higher abundance of respiratory chain complexes (C) in trained mice was further confirmed with western blots for components of CI–V (Figure 4h,i). In addition, the endothelial cell marker CD31 was more abundant in both running groups (Sed vs. Run, *p* = 0.0076; Sed vs. RR, *p* = 0.0004) indicating that both training modalities promoted capillarization (Figure 4j,k).

**FIGURE 5:**
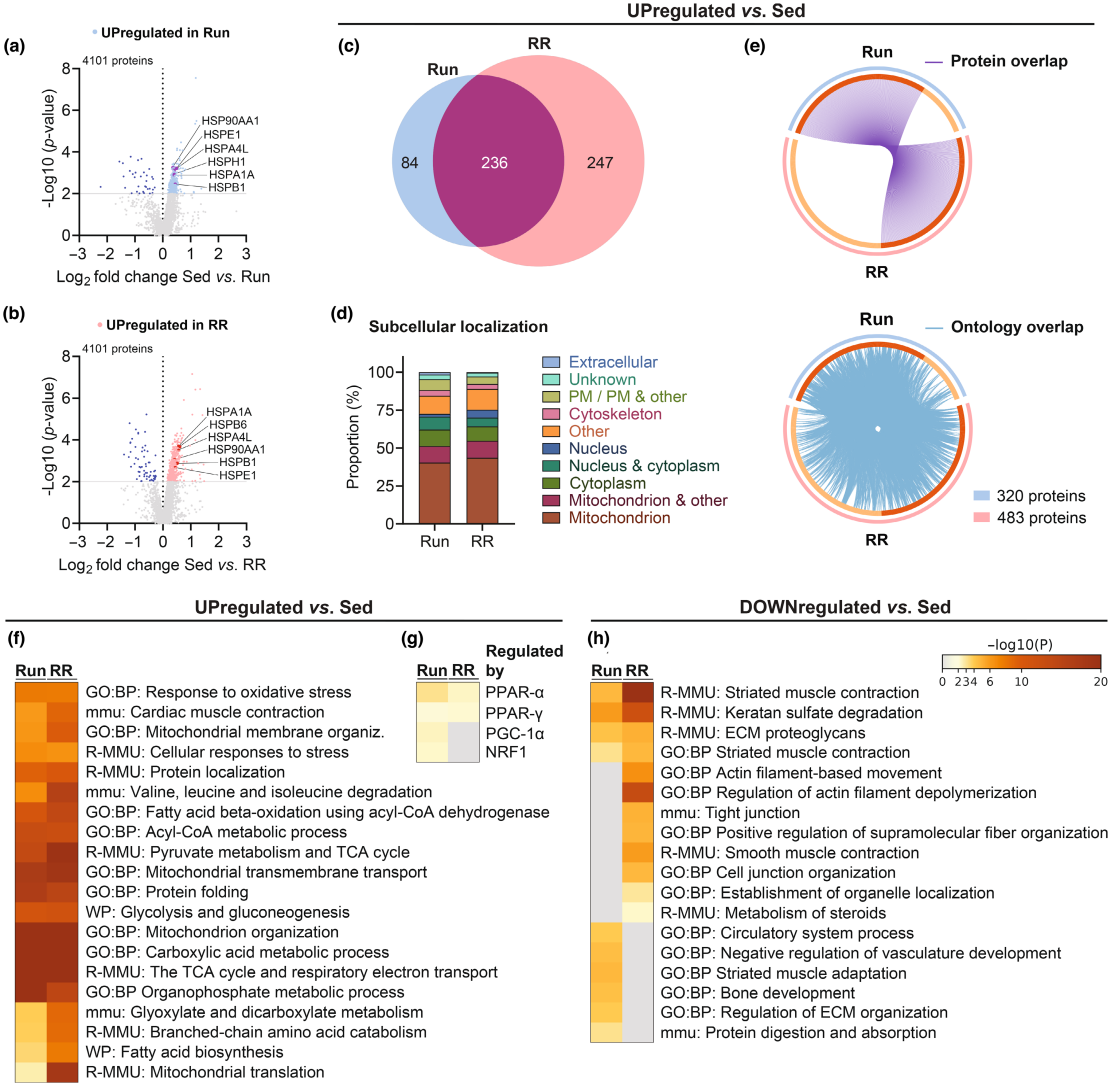
Proteomic adaptations of the *M. plantaris*. (a, b) Volcano plots showing the differentially regulated proteins in Run and RR compared to sedentary mice. (c) Venn's diagram showing the overlap between upregulated (*p*_unadjusted_ < 0.01) proteins in Run and RR compared to Sed along with (d) their UniProt‐based subcellular localization (PM: plasma membrane) and (e) Circos plots connecting the same proteins (purple lines) or proteins belonging to the same ontology term (blue lines). Heatmaps generated with Metascape showing (f) the top enriched ontology terms of upregulated proteins and (g) transcription factors/coregulators regulating these proteins as well as (h) top enriched ontology terms of significantly (*p*_unadjusted_ < 0.01) downregulated proteins. *n* = 4 for all datasets.

To obtain deeper insights into the molecular characteristics of *M. plantaris*, we performed mass spectrometry (MS)‐based proteomics. Among the 4101 detected proteins, 320 and 483 were upregulated in Run and RR, respectively (cutoff: *p*_unadjusted_ < 0.01) (Figure 5a–c and Tables S1–S3). Both training groups showed a robust basal accumulation of heat shock proteins (HSPs) (Figure 5a,b), an essential characteristic of trained skeletal muscle in rodents and humans (Amar et al., 2022; Mancini et al., 2019). The proteins enriched in the RR group recapitulated to a large extent the pattern in the Run animals (an overlap of 236 proteins out of 320 in total) besides an even larger group of proteins that was exclusively found to be enriched in RR (247 proteins) when compared to sedentary controls (Figure 4c). However, functionally, even these additional proteins were related to those in the Run animals (Figure 5e). Compared to sedentary mice, muscles of both Run and RR substantially elevated proteins located in mitochondria and cytoplasm (Figure 5d). Ontology enrichment analysis further confirmed a strong upregulation of proteins involved in mitochondrial organization, mitochondrial transmembrane transport, the citric acid (TCA) cycle, and respiratory electron transport in both groups, with a higher elevation of the anabolic processes fatty acid biosynthesis and mitochondrial translation in the RR mice (Figure 5f and Table S6). Regulatory factors linked to the change in protein levels included the peroxisome proliferator‐activated receptors α and у (PPARα/у) in both groups and the PPARγ coactivator 1α (PGC‐1α) as well as the nuclear respiratory factor 1 (NRF1) preferentially in the Run group (Figure 5g and Table S8). Surprisingly, despite the higher predicted activity of these two key regulators of mitochondrial biogenesis and oxidative metabolism, respiratory chain proteins were more consistently increased in RR mice, even though overall, the *M. plantaris* seems to adapt similarly to both training interventions in regard to mitochondrial oxidative phosphorylation. The boosted oxidative metabolism seemed to be at the expense of intra‐ and extracellular structural proteins as indicated by the ontology terms enriched among the downregulated proteins in both training groups (Figure 5h and Tables S4–S7).

### 3.4 Adaptations of the *M. soleus* in mice subjected to 10 weeks of Run or RR

**FIGURE 6:**
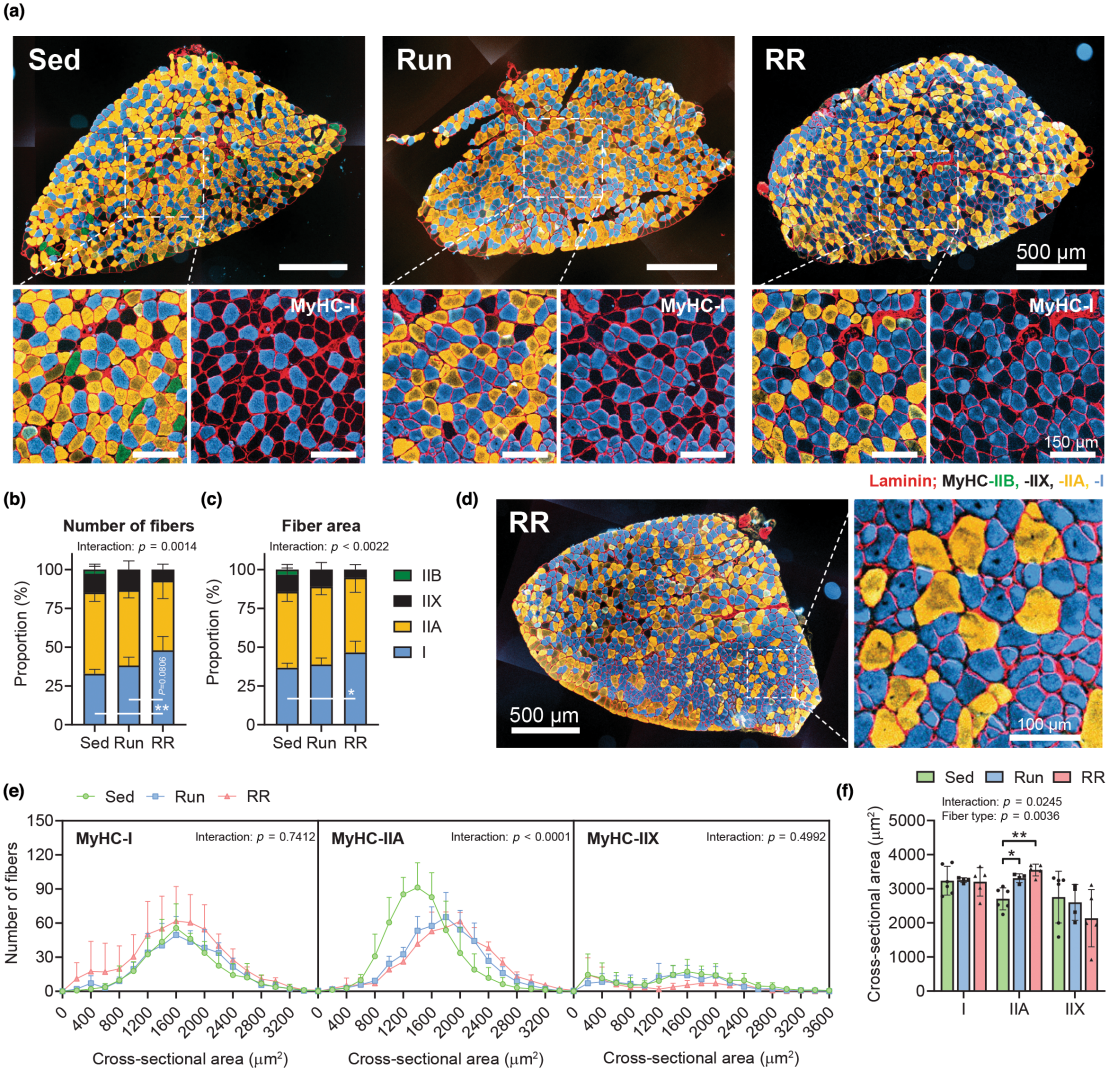
Cellular adaptations of the *M. soleus* following Run and RR. (a) Representative images from *M. soleus* whole‐muscle cross sections and further magnifications stained with antibodies against MyHC‐I (blue), ‐IIA (yellow), ‐IIB (green) as well as laminin (red), while fibers without staining were classified as MyHC‐IIX. Quantification of the MyHC‐isoform‐based distribution as a proportion of (b) the total number of fibers and (c) the total cross‐sectional area (CSA) occupied by fibers. (d) Whole‐muscle cross section and further magnification of an RR *M. soleus* sample that showed signs of MyHC‐I and ‐IIA hyperplasia. (e) Frequency histograms for the individual fiber types display the number of fibers that had the indicated CSA (μm^2^). (f) Mean CSA of MyHC‐I, ‐IIA, and ‐IIX fibers. (b, c, e, f) Sed, *n* = 7; Run, *n* = 5; RR, *n* = 6). Data are presented as mean ± SD including individual values where indicated. Two‐way ANOVAs followed by Tukey's post hoc tests (b, c, e, f). *p < 0.05, **p < 0.01.

The *M. soleus* was the only measured muscle that gained mass following training (Figure 3) and the stronger growth response observed in the RR group suggested a divergent adaptation to the two training modalities in this muscle. Indeed, besides the disappearance of the rare MyHC‐IIB fibers that occurred in both training groups, only RR mice elevated the proportion of MyHC‐I fibers (Sed vs. RR, *p* = 0.0014) and thus displayed a much stronger MyHC‐based faster to slower fiber type shift (Figure 6b). Similarly, MyHC‐I fibers occupied a larger proportion of the total fiber CSA only in RR mice (Sed vs. RR, *p* = 0.0165) (Figure 6c). Interestingly, some mice, especially in RR, showed signs of hyperplasia of MyHC‐I and in some cases MyHC‐IIA fibers, as indicated by relatively small fibers adjacent to a larger fiber with a centralized nucleus (black dot) (Run in Figure 6a and RR in Figure 6d). As both running groups experienced similar growth in MyHC‐IIA fiber size (Sed vs. Run, *p* = 0.0106; Sed vs. RR, *p* = 0.0015) (Figure 6e,f), the stronger increase in the number of MyHC‐I fibers in some RR mice could thus in part be responsible for the difference in *M. soleus* mass between Run and RR (*p* = 0.0307) (Figure 3).

**FIGURE 7:**
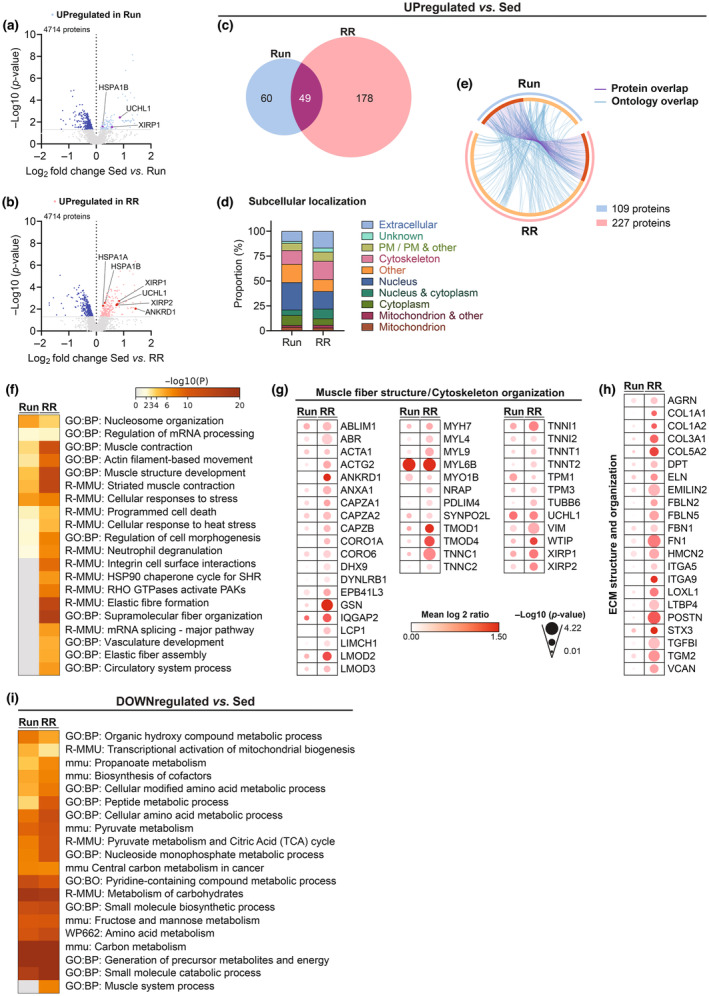
Proteomic adaptations of the *M. soleus*. (a, b) Volcano plots showing the differentially regulated proteins in Run and RR compared to sedentary mice. (c) Venn's diagram showing the overlap between upregulated (*p*_unadjusted_ < 0.05) proteins in Run and RR compared to Sed along with (d) their UniProt‐based subcellular localization (PM: plasma membrane) and (e) Circos plot connecting same proteins (purple lines) or proteins belonging to the same ontology term (blue lines). (f) Heatmap generated by Metascape showing the top enriched ontology terms of the upregulated proteins. Relative protein expression of selected proteins detected with TMT‐LC‐MS/MS involved in (g) Muscle fiber structure/cytoskeleton organization and (h) Extracellular matrix (ECM) structure and organization. (i) Heatmap generated by Metascape showing the top enriched ontology terms of significantly (*p*_unadjusted_ < 0.05) downregulated proteins in Run and RR. *n* = 4 for all datasets.

**FIGURE 8:**
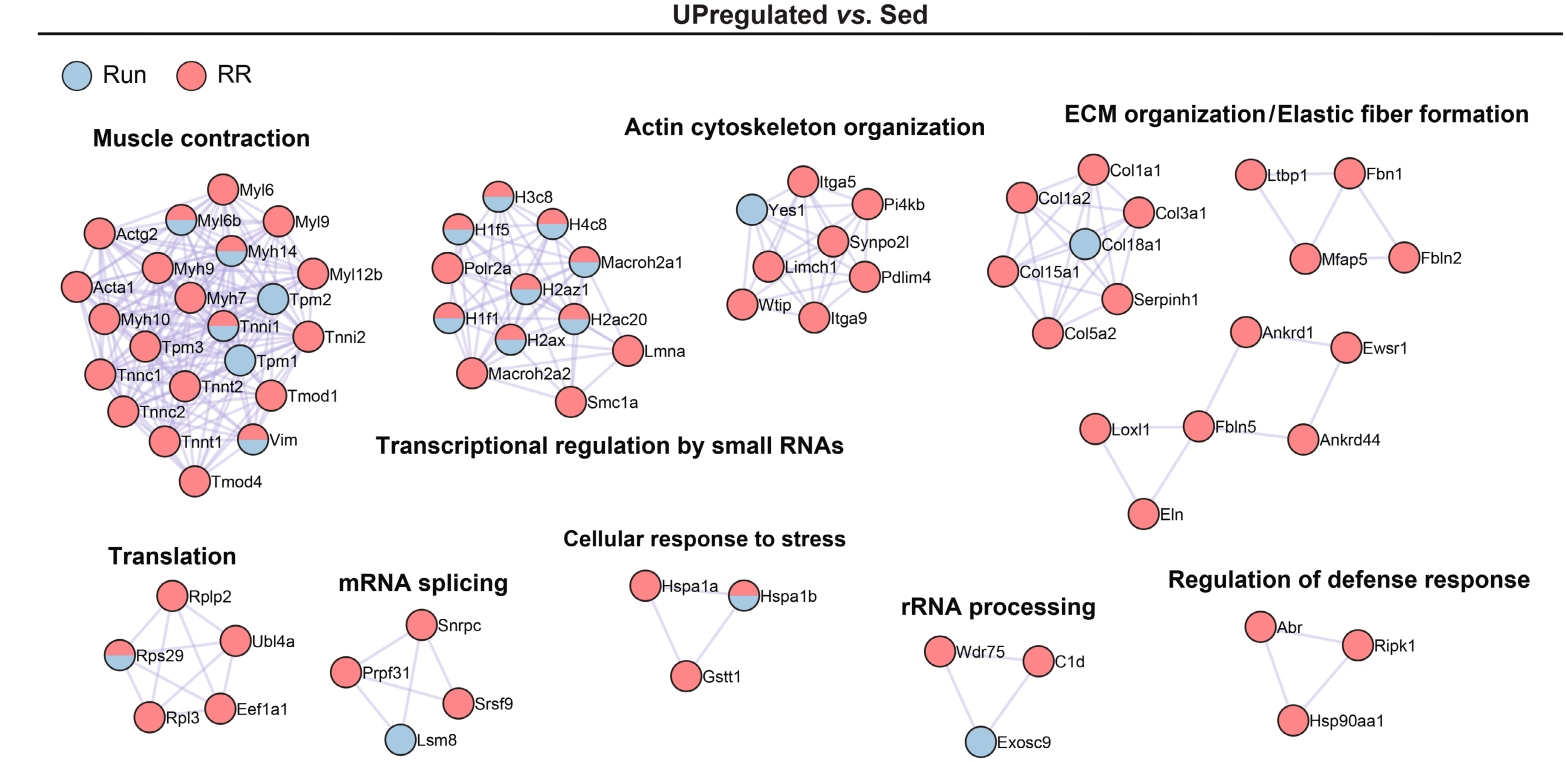
Protein–protein interaction networks of enriched proteins of Run and RR *M. soleus*. Metascape‐based protein–protein interaction enrichment analysis of significantly (*p*_unadjusted_ < 0.05) upregulated proteins in *M. soleus* of Run and RR compared to Sed. Displayed are the most densely connected networks with their individual components and functional description. *n* = 4 for all datasets.

Like the *M. plantaris*, we subjected the *M. soleus* to MS‐based proteomics for a detailed assessment of changes in the proteome in response to the two training modalities. The number of upregulated proteins in Run and RR compared to Sed was not as high as in the *M. plantaris* despite considering an even less stringent cut‐off (*p*_unadjusted_ < 0.05) (Tables S9–S11). In fact, downregulated proteins outnumbered the upregulated proteins in both Run (332 vs. 109) and RR (425 vs. 227) (Figure 7a,b; Tables S12 and S13). Ontology enrichment analyses revealed that these downregulated proteins were primarily linked to metabolic remodeling (Figure 7i and Table S15). In further contrast to the *M. plantaris*, a much smaller overlap between the Run and RR group was observed (Figure 7c) and mitochondrial proteins were largely absent in the proteins enriched in trained muscle (Figure 7d). In line, the top enriched ontology clusters did not contain terms related to oxidative metabolism and were more strongly or exclusively enriched in RR mice (Figure 7f and Table S14). Moreover, protein–protein interaction enrichment analysis revealed that RR muscles provided a much larger set of components in all of the most densely connected networks than Run muscles (Figure 8). Ankyrin repeat domain 1 (ANKRD1), a mechanical stress‐induced gene and yes‐associated protein (YAP, Hippo pathway) target, which is enriched upon hypertrophy stimulating resistance exercise in mice and humans or overload (Chaillou et al., 2013; Cui et al., 2020; Goh et al., 2019; Murach et al., 2022; Vissing & Schjerling, 2014), was exclusively found in RR samples (Figure 7b,g; Tables S11 and S16). In contrast, several proteins whose mRNA expression was highly induced upon an acute hypertrophic training stimulus in mice (xin actin‐binding repeat‐containing protein 1 [XIRP1], ubiquitin carboxyl‐terminal hydrolase isoenzyme L1 [UCHL1], and heat shock 70 kDa protein 1 A and B [HSPA1A and HSPA1B]; Cui et al., 2020) were similarly enriched in both groups except for HSPA1A, which was only upregulated following RR (Figure 7a,b,g; Tables S10, S11, and S16). Expression of agrin (*Agrn*), which plays a major role in the formation and maintenance of the neuromuscular junction and potentially the promotion of capillarization (Bezakova & Ruegg, 2003; Chakraborty et al., 2020), was enriched and its promoter CpG hypomethylated in myonuclear DNA in the *M. soleus* of older mice trained with weighted wheel running (Dungan et al., 2022). In our dataset, the agrin protein (AGRN) was upregulated in the muscles of both trained groups (Figure 7h and Table S16).

Many of the top ontology terms and the relative enrichment of cytoskeleton and extracellular proteins (Figure 7d,f) indicated intra‐ and extracellular structural remodeling as a core adaptation of *M. soleus* to running wheels. However, with the applied cut‐off, most of the proteins involved in these processes seemed to be more strongly enriched in RR, even though they showed a similar trend also in low‐resistance wheel running (Figure 7g,h and Table S16). Besides components of the sarcomere, XIRP 1, XIRP 2, nebulin‐related anchoring protein were among the top upregulated proteins in RR (Figure 7g and Table S16). These peptides are present in the myotendinous junction (MTJ) as well as costameres, two major anchoring structures involved in force transmission (Henderson et al., 2017). In addition, the reactome pathway Rho GTPase‐activated p21‐activated kinases (PAKs) was enriched only in RR (Figure 7f; Tables S12 and S14). PAKs are a family of serine/threonine kinases implicated in cytoskeletal rearrangements and Rho GTPases have been linked to muscle mass regulation, while being upregulated upon load‐induced hypertrophy (Murach et al., 2022; Rodriguez‐Fdez & Bustelo, 2021).

**FIGURE 9:**
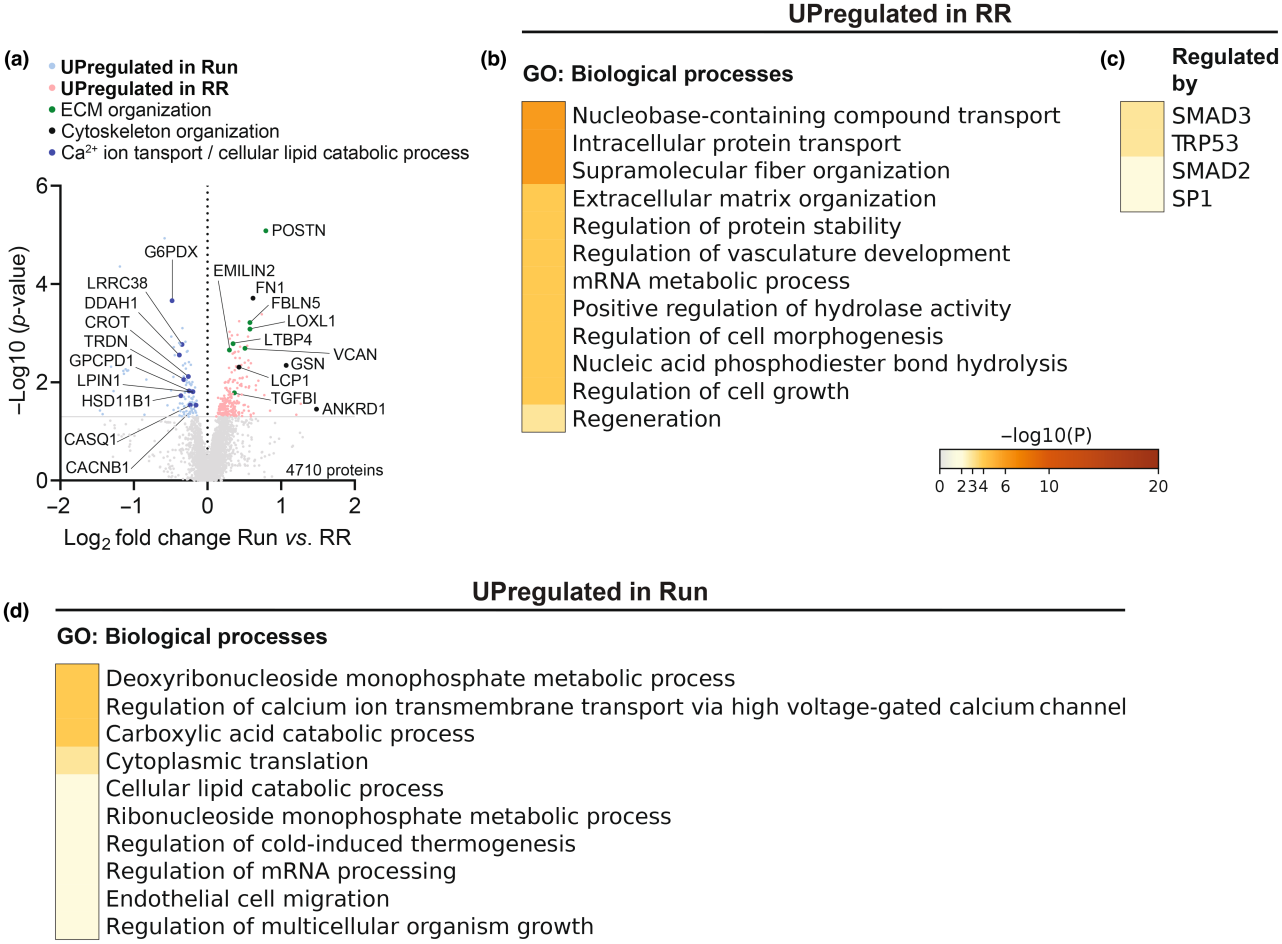
Direct comparison between Run and RR of TMT‐LC‐MS/MS detected proteins of the *M. soleus*. (a) Volcano plot showing differentially regulated proteins of Run compared to RR (*n* = 4), light blue and salmon mark proteins upregulated (*p*_unadjusted_ < 0.05) in Run or RR, respectively. Selected proteins are labeled and colored according to the indicated functions. Metascape's top GO: Biological processes of the upregulated proteins in (b) RR and (d) Run along with transcription factors predicted to regulate the enriched proteins in RR in (c).

Finally, the direct comparison of the proteins in Run and RR muscles revealed a selective presence of extracellular matrix (ECM) organization proteins in the latter group (Figure 9a,b and Tables S17–S20). Among those, periostin (POSTN), fibulin 5 (FBLN5), lysyl oxidase homolog 1 (LOXL1), latent‐transforming growth factor beta‐binding protein 4 (LTBP4), versican core protein (VCAN), emilin‐2 (EMILIN2), and transforming growth factor‐beta‐induced (TGFBI), all key components and/or regulators of the ECM and connective tissue architecture were most robustly enriched (Figure 9a and Table S18). Furthermore, important elements and regulators of the cytoskeleton were strongly different from Run such as fibronectin (FN1), gelsolin (GSN), plastin‐2 (LCP1 or PLS2), and ANKRD1 (Figure 9a and Table S18). TRRUST enrichment analysis predicted the transcription factors small worm phenotype/mothers against decapentaplegic (SMAD) 2 and 3, tumor protein P53 (TRP53), and specificity protein 1 (SP1) as regulators of the RR specific proteome (Figure 9c and Table S22). In contrast, proteins upregulated in Run in this comparison have been implicated in regulating calcium ion transmembrane transport and catabolic processes of carboxylic acids and cellular lipids (Figure 9d and Table S21).

## 4 DISCUSSION

Exercise adaptations arise from a complex interplay of different stimuli and perturbations, which are strongly associated with the corresponding training protocol. In human athleticism, many disciplines, and hence the respective training approaches, require both endurance and resistance components to varying degrees. Thus, strategies have to be found to maximize performance while minimizing potential interference effects in combined or concurrent training (Fyfe et al., 2014). The most commonly used trial‐and‐error approach to devise such strategies optimally would be replaced by—or at least combined with—mechanistic insights to allow evidence‐based planning of personalized, efficient, and safe training for athletes and non‐athletes alike. To a large extent, due to the inherent limitations of human studies, such data on signaling pathways, molecular mediators, and biomarkers for training progression are obtained in model systems. However, to ensure translatability, a comprehensive understanding of these models is imperative. In this investigation, we, therefore, have comprehensively assessed endurance and grip strength performance as well as muscle morphological and proteomic adaptations of mice exposed to voluntary resistance running wheels. Moreover, we have compared these results to those obtained with low‐resistance wheel running.

In essence, high‐ and low‐resistance running wheels resulted in shared, but also distinct outcomes. Thus, high‐resistance wheel running recapitulated aspects of endurance training adaptations triggered by free running wheels, for example, related to *V̇*O_2peak_, RER, post‐exercise lactate levels, capillarization, or mitochondrial protein levels. However, parameters such as the reduced submaximal endurance performance of RR compared to Run mice imply a diminished effect of high‐resistance wheels for overall endurance outcomes. Inversely, structural and functional markers of resistance training adaptation most strongly improved in the RR group, for example, muscle mass, grip strength, and relative lean mass, associated with a distinct proteome modulation. Thus, high‐resistance wheel running might represent a more hybrid form of training, conveying both endurance and strength gains.

Importantly, individual muscles react in distinct manners to both training modalities. For example, the *M. plantaris* showed a pronounced glycolytic to oxidative fiber type shift in both training groups but did not undergo any change in mass. In contrast, the *M. soleus* experienced hypertrophy following both high‐ and low‐resistance wheel running but a substantial MyHC‐based fiber type shift was only observed in RR mice. The higher proportion of MyHC‐I fibers in RR was potentially driven by hyperplasia, which could also in part explain the higher *M. soleus* mass in RR compared to Run despite a similar increase in MyHC‐IIA fiber CSA in both training groups. Note that based on the results, hyperplasia cannot conclusively be proven, and other events, for example, fiber splitting, ruled out. The *M. soleus* hypertrophy in response to low‐resistance wheel running is in agreement with some, but not all previous reports using the same (Masschelein et al., 2020) or other running wheels (Allen et al., 2001; Brooks et al., 2018; Englund et al., 2020; Jackson et al., 2015; Li et al., 2006). Whether the increase in mass translates into higher force production of the *M. soleus* in any or both of the training modalities remains to be tested. Moreover, whether Run and RR differentially affect satellite cell activation and myonuclear accretion, both important aspects in resistance training adaptation (Bagley et al., 2023), has to be addressed in future studies. However, the proteomic signatures of trained *M. soleus* and *M. plantaris* exhibited differences, and in both muscles, low‐ and high‐resistance wheel running have elicited distinct adaptations. These results have to be interpreted in light of the low stringency cut‐offs due to the difficult analysis of muscle tissue by MS. Despite this drawback, the data, complemented by the functional, morphological, and cellular results, help to interpret the training paradigm‐specific adaptations. Changes in the *M. plantaris* were mostly characterized by an increase in mitochondrial and oxidative metabolism proteins, whereas the *M. soleus* upregulated components of the cytoskeleton and the ECM. Interestingly, the structural protein changes appeared to be more complex and distinctive in the *M. soleus* of RR mice, while Run favored the elevation of proteins involved in calcium transport as well as carboxylic acid and lipid catabolic processes. In addition to its potential contribution to muscle mass, the cytoskeleton network within muscle transmits force both along the length of each muscle fiber and from the center to the outside of the fiber (Hughes et al., 2015). While there is limited data on the adaptations of cytoskeleton proteins to resistance training and their influence on muscle mass and strength gains (Hughes et al., 2018), costameric proteins, including those enriched in RR, are prominently downregulated in bed rest and spaceflight and are restored upon reloading (Murgia et al., 2022). Importantly, the decrease in costameric proteins in these reports correlated with the loss of muscle strength (Monti et al., 2021; Rittweger et al., 2018). Besides the reinforcement of costameres, MTJs, and the sarcomere itself, intact ECM remodeling might be critical for normal muscle growth and improved force generation in response to weighted wheel running (Brightwell et al., 2022; Englund et al., 2021). The study of key regulators of the cytoskeleton and ECM organization could therefore be fundamental for identifying the mechanisms responsible for the RR‐specific adaptations. Our proteomics analysis revealed several candidates, including POSTN, LOXL1, LTBP4, TGFBI, GSN, and LCP1 that could potentially mediate these effects. Future studies should, however, carefully validate the upregulation of these proteins in the high‐, as well as those that define the low‐resistance context. Further, whether the potentially broader cytoskeleton and ECM remodeling of the *M. soleus* is also observed in muscles more prominently involved in the grip strength task and therefore indeed confers the robust increase in grip strength performance to RR, has yet to be defined. Even though the RR‐specific increase in grip strength is in accordance with a study using a similar setup (Masschelein et al., 2020), it should be noted that others observed increased grip strength also following 8 weeks of wheel running without additional load (Kim et al., 2020). Of note, some of these proteins, for example, XIRP1, have also been detected in other contexts, in this case, the satellite cells as a marker of muscle damage and regeneration (Al‐Sajee et al., 2015; Nilsson et al., 2013). Such mechanisms could thus also contribute to the observed results. ANKRD1, which we found to be enriched only in the muscles of RR mice in the *M. soleus*, is another protein that could contribute to resistance training adaptations. ANKRD1 is a mechanical stress‐induced protein linked to muscle hypertrophy in different resistance exercise contexts in mice and humans, or in overload (Chaillou et al., 2013; Cui et al., 2020; Goh et al., 2019; Vissing & Schjerling, 2014). Based on these observations, it appears that RR may provide a physiological model to study such emerging regulators of muscle hypertrophy and potentially other resistance training adaptations. Moreover, different muscles might help in the investigation of various aspects of training adaptation to low‐ and high‐resistance running wheels.

From an exercise physiology perspective, the difference in running performance despite similar improvements in *V̇*O_2peak_ as well as capillarization and mitochondrial protein content in the *M. plantaris* is curious. The external work performed by the Run and RR groups was comparable, and thus, cardiovascular adaptations important for oxygen delivery, in many settings strongly determining *V̇*O_2peak_ (Bassett Jr. & Howley, 2000), might likewise be in the same range. This certainly seems to be the case for muscle capillarization. To improve submaximal endurance performance in response to training, muscle oxygen extraction, metabolic changes, and structural adaptations might be more critical, even if they do not lead to an increase in *V̇*O_2peak_ (Hawley et al., 2018). Even though both groups exhibited a broad increase in mitochondrial proteins after 10 weeks of training, the mitochondrial biogenesis regulators PGC‐1α and NRF1 were predicted to be active only in the Run, and not in the RR group. Notably, PGC‐1α also positively regulates myoglobin (Lin et al., 2002). Future studies will determine whether these seemingly subtle differences contribute to submaximal exercise performance. In addition, it is conceivable that Run mice were better habituated to longer and continuous running due to the larger daily distance and higher average speed compared to RR mice.

A potential limitation of this study is that only male mice were analyzed. Even though our results are, to a large extent, in accordance with a previous study using the same wheels and a similar experimental setup applied to female mice (Masschelein et al., 2020), it is possible that some of our findings do not fully translate to females. Therefore, to allow for a direct comparison of the commonalities and discrepancies in training adaptations between males and females, future studies should include both sexes.

## 5 CONCLUSIONS

Our data indicate that low‐ and high‐resistance wheel‐running interventions elicit overlapping, but also largely distinct outcomes in molecular, morphological, and functional training adaptations. Low‐resistance wheel running represents a modality leading to many of the described changes of endurance training, with the unexpected finding of *M. soleus* hypertrophy. High‐resistance wheel running might recapitulate the consequences of hybrid training, showing markers of endurance and resistance training adaptation. This model thus might be particularly useful for many disciplines in which both types of performance are important. Finally, the divergent response of individual muscles could further expand the study of different mechanisms of structural and functional remodeling. Thus, hopefully, our results will help in the design and selection of training interventions of animal models, and thereby support the discovery of factors and mechanisms mediating exercise adaptation.

## AUTHOR CONTRIBUTIONS

Conceptualization, Aurel B. Leuchtmann and Christoph Handschin; Methodology, Aurel B. Leuchtmann, Yasmine Afifi, Danilo Ritz, and Christoph Handschin; Formal Analysis, Aurel B. Leuchtmann, Yasmine Afifi, Danilo Ritz; Investigation, Aurel B. Leuchtmann, Yasmine Afifi, and Danilo Ritz; Data Curation, Danilo Ritz; Visualization, Aurel B. Leuchtmann; Writing—Original Draft, Aurel B. Leuchtmann; Writing—Review & Editing, Aurel B. Leuchtmann and Christoph Handschin; Supervision, Aurel B. Leuchtmann and Christoph Handschin; Project Administration, Aurel B. Leuchtmann; Funding Acquisition, Christoph Handschin.

## FUNDING INFORMATION

This research was supported by grants from the Swiss National Science Foundation (SNSF, grant 310030_184832), Innosuisse (grant 44112.1 IP‐LS), the Swiss Society for Research on Muscle Diseases (SSEM), the Jain Foundation, the Novartis Stiftung für Medizinisch‐Biologische Forschung and the University of Basel.

## CONFLICT OF INTEREST STATEMENT

The authors declare no competing interests.

## ETHICS STATEMENT

All procedures involving animals were performed in accordance with Swiss regulations for animal experimentation, approved by the veterinary commission of the Canton Basel‐Stadt (Switzerland) [reference numbers: 2329 (cantonal), 29587 (national)] and performed in accordance with the Swiss federal guidelines for animal experimentation under consideration of the wellbeing of the animals and the 3R (replace, reduce, and refinement) principle. The manuscript does not contain clinical studies or patient data.

## Supporting information


Tables S1–S8.

Tables S9–S16.

Tables S17–S22.
